# Prognostic Value of the Echocardiographic Probability of Pulmonary Hypertension in Patients with Acute Decompensated Heart Failure

**DOI:** 10.3390/jcm8101684

**Published:** 2019-10-15

**Authors:** Sebastian Carballo, Philippe Musso, Nicolas Garin, Hajo Müller, Jacques Serratrice, François Mach, David Carballo, Jérôme Stirnemann

**Affiliations:** 1 Service of Internal Medicine, Department of Medicine, Geneva University Hospitals, Geneva 1211, Switzerland; 2 Service of Cardiology, Department of Medicine, Geneva University Hospitals, Geneva 1211, Switzerland

**Keywords:** heart failure, pulmonary hypertension, echocardiography

## Abstract

The prognostic value of pulmonary hypertension (PH) estimated by echocardiography in unselected patients with acute decompensated heart failure (ADHF) is poorly studied. Between November 2014 and September 2018, 657 patients were recruited in a prospective registry of ADHF (ClinicalTrials.gov NCT02444416). The probability of pulmonary hypertension was based on European Society of Cardiology (ESC) guidelines for echocardiographic evaluation. The median survival without all-cause mortality or readmission was 7 months. During the median follow-up period of 15 months, there were 450 events including 185 deaths. In multivariate analysis, the hazard ratio (HR) of all-cause mortality or readmission for patients with a high probability of PH was 1.67 (95% CI 1.29–2.17, *p* < 0.001) as compared to patients with a low or intermediate probability. The left ventricular ejection fraction (LVEF) and right ventricular function (RVF) were not associated with the primary outcome—HR 1.02 (95% CI 0.81–1.29; *p* = 0.84) and 0.96 (95% CI 0.76–1.23; *p* = 0.77) respectively. In patients admitted for ADHF, a high probability of PH as evaluated by echocardiography provided the highest independent prognostic value for mortality and readmission, whereas LVEF and RVF were not associated with prognosis. The identification of patients at high risk of PH by non-invasive measurement conveys important prognostic information and may guide management.

## 1. Introduction

Despite the availability of numerous therapeutic agents and advances in patient management, prognosis remains poor in heart failure (HF) [1,2]. Acute decompensated heart failure (ADHF) is a leading cause of hospitalizations and mortality in patients older than 65 years [3,4]. Prognosis of HF depends on etiology as well as on factors such as age, renal function, blood pressure, left ventricular ejection fraction (LVEF), brain natriuretic peptide (BNP) levels and certain comorbidities [5,6,7,8]. The prognostic value of pulmonary hypertension (PH) as estimated by echocardiography in unselected patients with acute decompensated heart failure (ADHF) has previously been addressed in small or pilot studies with <1 year follow-up, suggesting incremental prognostic information and ominous prognosis [9,10]. Cardiac function, and in particular left ventricular ejection fraction (LVEF) has a contrasting prognostic value [11]. Furthermore, HF with reduced ejection fraction (HFrEF) and HF with preserved ejection fraction (HFpEF) have a similar prognosis [12]. The added prognostic value of right ventricular function (RVF) and pulmonary artery pressure (PAP) has been evaluated in chronic HFrEF and HFpEF, but with diagnostic techniques seldom available in standard management settings of patients admitted with ADHF [13,14,15,16,17,18,19]. RVF is sensitive to afterload and its prognostic value may in fact be intimately linked to PAP and particularly to PH [14,19]. The importance of these parameters in patients admitted with ADHF is unclear. European Society of Cardiology (ESC) guidelines for the diagnosis and treatment of PH state that echocardiography should always be performed when PH is suspected [20]. The purpose of this study was to further evaluate the prognostic value of the echocardiographic probability of PH, independently of LVEF and RVF, as evaluated by standard echocardiography in patients with ADHF.

## 2. Methods

### 2.1. Patients

Between November 2014 and September 2018, consecutive patients with ADHF admitted to the Department of medicine were recruited in a prospective registry of acute heart failure at the University Hospitals of Geneva (ClinicalTrials.gov NCT02444416). The study included patients with symptoms of heart failure based on the European Society of Cardiology (ESC) definition, such as dyspnea, ankle swelling and fatigue, accompanied by signs of heart failure such as elevated jugular venous pressure, pulmonary crackles or peripheral edema due to structural and/or functional cardiac abnormality [6]. Additional inclusion criteria were elevated brain natriuretic peptide (BNP) levels >100 ng/L, or pro-BNP levels >300 ng/L. All patient data were reviewed by a senior investigator, with expertise in the diagnosis and management of heart failure. The protocol was approved by the institutional ethics committee (protocol CER 14-019), and all patients gave written informed consent.

Information, including age, gender, weight, smoking status, a full medical history and medical therapy at admission, throughout hospital stay and at discharge were recorded. Clinical presentation, including New York Heart Association (NYHA) dyspnea class, and clinical parameters, such as blood pressure, heart rate, and weight, were also recorded at admission and throughout hospital stay. Customary investigations were carried out on all patients, including full blood count, urea and electrolytes, electrocardiography, and echocardiography.

Outcomes were collected at 3, 12 and 24 months, and yearly afterwards, and included mortality, readmission, clinical state, and medication. Follow-up was carried out through contact with treating physicians and examination of hospital medical records.

### 2.2. Echocardiography

Echocardiography was analyzed by experienced staff cardiologists. Left and right cardiac chamber characteristics were recorded, and patients were classified according to ESC guidelines as having heart failure with either HFrEF if LVEF was <40%, mid-range ejection fraction (HFmrEF) if LVEF was between 40% and 49%, or HFpEF if LVEF was ≥50%, using both elevated natriuretic peptides and relevant structural heart disease or diastolic dysfunction as additional criteria [6]. Right ventricular function (RVF) was defined as normal if tricuspid annular plane systolic excursion (TAPSE) was ≥16 mm and pulsed wave tissue Doppler velocity of the tricuspid annulus (s’) ≥9 cm/s. RVF was also defined as normal if one of either TAPSE or s’ recording was below the respective threshold, but radial function was normal. RVF was defined as decreased for TAPSE <16 mm and s’ <9 cm/s, or if one of either TAPSE or s’ values was above their respective threshold but radial function was decreased. The probability of pulmonary hypertension was based on European Society of Cardiology (ESC) guidelines echocardiographic evaluation [20]. Low probability was defined as peak tricuspid regurgitation velocity (PTRV) ≤2.84 m/s or not measurable, and no other echocardiography signs of PH; intermediate probability was defined as PTRV ≤2.84 m/s or not measurable with other signs of PH, or PTRV 2.85–3.4 m/s and no other echocardiography signs of PH; high probability was defined as PTRV 2.85–3.4 m/s with other signs of PH, or PTRV >3.4 m/s. Other signs of PH were end systole right atrial area >18 cm^2^ and flattening of the interventricular septum (left ventricular eccentricity index >1.1 in systole and/or diastole, defined as the ratio of the anterior—inferior and septal—posterolateral cavity dimensions at the mid-ventricular level). As both low and intermediate probability can be estimated even with low or non-measurable PTRV, two categories were used for analysis: a low and intermediate probability of HP versus a high probability of PH.

### 2.3. Study Outcomes

The primary outcome was all-cause mortality or readmission. Secondary outcomes were all-cause mortality, cardiovascular death or readmission, and cardiovascular mortality. Death or readmission were considered as being of cardiovascular origin if related to heart failure, ischemic heart disease or stroke.

### 2.4. Statistical Analysis

We used median (interquartile range) for continuous data, and number (%) for categorical data, as appropriate. Student or Mann–Whitney–Wilcoxon tests were used for comparing quantitative data, and Chi-square or Fischer tests for categorical comparisons. The distribution of these variables was tested, and non-parametric tests were used when the distribution was not normal. For the study outcomes, we used survival analysis with Log-Rank test for the unadjusted analysis, Kaplan–Meier plots, and Cox proportional hazard models for multivariate exploration. The multivariate model included variables that were associated with outcome on unadjusted analysis (*p* < 0.02), as well as commonly recognized variables identified in previous studies. Patients for whom follow-up was not available were censored. Statistical analysis was performed using the R statistical software package, version 3.1.1 (www.cran.r-project.org).

## 3. Results

### 3.1. Baseline Characteristics

Between November 2014 and September 2018, 657 consecutive patients were included. The median age was 78.7 years; 117 patients had a high probability, and 540 had a low or intermediate probability of PH. Baseline characteristics were comparable across both groups (Table 1). In patients with a high probability of PH, proportionally more patients had a reduced RVF as compared to those with a low or intermediate probability of PH, with no significant difference for left ventricular function. Clinical, comorbidities and biological parameters at admission were similar in both groups, including NYHA class, blood pressure, and BNP levels. There was no significant difference in gender or BMI. With respect to medication at admission, proportionally more patients with a high probability of PH had loop diuretics (Table 2). There were five patients lost to follow-up.

The median follow-up period in this cohort was 15 months (interquartile range (IQR) 6–24 months); there were 450 events during this period including 185 deaths. The median survival without death or readmission was 7 months (IQR 5–9 months).

### 3.2. Association between LVEF, RVF and PH and All-Cause Mortality or Readmission

On univariate analysis, a high probability of PH, a history of COPD, chronic kidney disease and anemia were associated with a higher risk of of-cause death or readmission, whereas age and BMI were protective (Table 3). LVEF was not associated with outcome. Of the left-sided valvular diseases, both aortic stenosis (AS) and mitral stenosis (MS) were associated with the outcome.

LVEF, RVF and probability of PH were included in the multivariate model, as well as known aetiologias of PH such as left-sided valvular disease (AS and MS) or a history of COPD. Chronic kidney disease, anemia, age and gender were also included either because of significant association on univariate analysis, or because they are established predictors of outcome (Table 4) [7].

**Figure 1:**
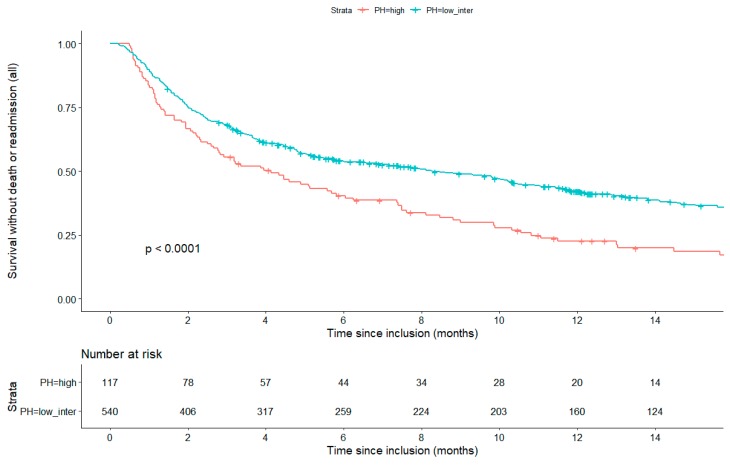
Kaplan–Meier survival analysis for the primary outcome of all-cause mortality and readmission in patients with a high versus a low or intermediate probability of pulmonary hypertension (PH).

On multivariate analysis, the probability of PH was significantly associated with a poor outcome. The hazard ratio of all-cause mortality or readmission in patients with a high probability of PH was 1.67 (95% CI 1.29–2.17; *p* < 0.001) as compared to patients with a low or intermediate probability of PH (Table 4). LVEF and RVF were not associated with the primary outcome with hazard ratios of 1.02 (95% CI 0.81–1.29; *p* = 0.84) and 0.96 (95% CI 0.76–1.23; *p* = 0.77) respectively. Aortic stenosis and mitral stenosis were also associated with poor outcome, with HR’s of 1.5 (95% CI 1.06–2.12, *p* = 0.02) and 2.37 (95% CI 1.2–4.66, *p* = 0.01) respectively, as was a history of COPD (HR 1.47 (95% CI 1.13–1.91; *p* = 0.004), and chronic anemia (HR 1.31 (95% CI 1.06–1.62; *p* = 0.01). Of note, there was no significant difference in the proportion of patients with a history of COPD in the two PH groups (Table 1). Interaction variables with history of COPD × PH, aortic stenosis × PH, and mitral stenosis × PH were tested in a multivariate Cox model and were non-significant. Furthermore, interaction variables of BNP × LVEF, BNP × RVF and BNP x PH were also tested in a multivariate Cox model and were non-significant. When considering all-cause mortality, a high probability of PH was also significantly associated with a poor outcome—HR 2.04 (95% IC 1.38–3.01, *p* < 001) (Figure 1).

The probability of PH was also significantly associated with the secondary outcomes, including worse cardiovascular mortality or readmission. The hazard ratio of cardiovascular mortality or readmission in patients with a high probability of PH was 2.04 (95% CI 1.38–3.01, *p* < 0.001), as compared to patients with a low or intermediate probability of PH (Table 4). LVEF and RVF were not associated with this outcome, with hazard ratios of 1.13 (95% CI 0.86–1.48, *p* = 0.38) and 1.07 (95% CI 0.81–1.40, *p* = 0.65) respectively. Mitral stenosis and a history of COPD were also associated with poor outcome. These observed associations were similar when considering cardiovascular mortality alone.

## 4. Discussion

In this prospective cohort of patients admitted for ADHF, a high probability of PH as evaluated by echocardiography was strongly and independently associated with all-cause and cardiovascular mortality or hospital readmission. The evaluation of PH therefore appears to be particularly important in the initial appraisal and management of patients with ADHF. Furthermore, our study prospectively evaluated PH using routinely available techniques in patients admitted with ADHF.

The additive prognostic value of the echocardiographic estimation of PH in patients with HF is poorly studied and not integrated into current management guidelines of ADHF [6,7,8,21]. Whilst left ventricular function and, more recently, RVF have been shown to be associated with mortality, the relationship between these parameters and prognosis is not systematically demonstrable and the clinical applicability of certain measurement modalities is limited, as acknowledged by American Heart Association and European Heart Society guidelines [6,8,11,12,21,22]. Although the independent prognostic value of RVF and PAP has been described, this has been in selected populations, such as patients with idiopathic dilated cardiomyopathy and often in the context of heart transplantation evaluation, or with invasive right heart catheterization [14,23,24]. There is a demonstrated inverse relationship between PAP and RVF, although only patients with a high PAP and low RVF have reduced survival [14]. In cases of PH attributed to both a postcapillary component and a superimposed precapillary component, termed “reactive PH” by the authors, a mean PAP >25 mmHg is associated with a HR of 7.6 for all-cause mortality at 6 months [23]. The impact of RVF and PAP coupling has also been described in a community cohort regarding the risk of developing heart failure but the prognostic value of PAP was not addressed [19]. The effects of RVF were also described retrospectively in a cohort of patients with chronic systolic heart failure [18]. In this study, RVF was measured using gated-equilibrium radionuclide ventriculography, PAP was not evaluated, and right ventricular ejection fraction <20% was found to be a significant independent predictor of mortality and hospitalization. Of note, the prognostic value of LVEF is not linear and subject to several confounders [11,12].

PH is often multifactorial, but PH associated with left heart disease is the most common form, accounting for up to 80% of cases and is referred to as World Health Organization pulmonary hypertension group 2 [25,26,27,28]. PH can be present in patients with both HFrEF and HFpEF, with the common underlying feature being a chronic increase in left atrial pressure [25]. In addition, nitric oxide-dependent pulmonary vasodilation may be impaired in HF, further contributing to a reactive PH [25]. Left valvular disease often leads to the development of PH, albeit with variability in pulmonary vascular responses, and PH is an important prognostic element in these patients [25,29]. The gold standard for evaluating PH remains cardiac catheterization, in particular when specific treatment for pulmonary arterial hypertension is being considered. PH is defined by a mean PAP >25 mmHg, a left heart component to PH is considered when pulmonary capillary wedge pressure >15 mmHg or left ventricular end-diastolic pressure >18 mmHg [25]. However, the invasive nature of catheterization, and the number of HF patients and hospitalizations make this type of measurement impractical in routine care. In the clinical setting, echocardiography remains a readily available tool. Our study demonstrates that standard values obtained from echocardiography can robustly predict poor outcome related to PH. In our cohort, the proportion of patients with valvular disease, principally mitral regurgitation and aortic stenosis, was similar in both groups of PH patients, namely a high probability and a low or intermediate probability of PH. Aortic stenosis and mitral stenosis were associated with all-cause mortality or readmission. PH due to lung disease and or hypoxia constitutes the WHO group 3 of PH [30]. In our study, no significant difference in the proportion of patients with a history of COPD was observed between PH groups. Interestingly, a history of COPD was associated with outcomes. HF and COPD share common risk factors and whilst the prevalence of concurrent HF and COPD vary, most studies suggest a rate of between 10% and 40% [31,32]. As apparent in our study, concurrent COPD has previously been shown to independently predict mortality in patients with HFrEF as well as HFpEF [31,33].

The close interaction between LVEF, the development of PH, and RVF is well described, and therefore it could be assumed that the prognostic value of each of these parameters would show a degree of overlap or co-linearity of association. Previous studies have only investigated selected HF patient populations; and furthermore, PH has only been assessed as an independent marker of prognosis only in specific cases such as reactive PH [23]. Our study suggests that when LVEF, RVF and echocardiographic evaluation of PH are simultaneously analyzed in non-selected patients with ADHF, PH is the strongest predictor of poor outcome. Of note, preserved and mid-range LVEF were similar in both patient groups, irrespective of estimation of PH. There was no statistical difference for decreased LVEF in the two PH groups, was present in 24% of patients in the high probability group versus 33.1% in the low or intermediate group (*p* = 0.05). In addition to the fact that LVEF was not significantly associated with outcome on multivariate analysis, this finding confirms the complex relationship between LVEF and prognosis [11].

The management of HF, and in particular ADHF, is complex and is well codified for HFrEF—less so for HFpEF. Estimation of prognosis is equally difficult and dependent on intrinsic cardiac properties as well as comorbidities. The clinical implications of this study are that the routine evaluation of PH and its incorporation into the care of patients admitted with ADHF may enable improved risk stratification, particularly for patients with a high probability of PH. Attention to valvular disease and specific therapies for PH such as the appropriate use of diuretics and monitoring may be important in this group.

Our study has limitations. First, because of their observational nature, our findings are limited to association and not causality. Furthermore, despite multivariate analysis, there is residual confounding. Second, data collection on past medical history was based on medical records. In the case of COPD, cross-reference with pulmonary function results was not carried out, which may have led to the misclassification of this exposure risk. The strengths of our study are its prospective design, the consecutive inclusion of patients admitted with ADHF, and the use of robust clinical and biological inclusion criteria based on accepted guidelines and definitions for the identification of patients with HF [6,8,34,35]. Patients with HF but with no acute decompensation or PH were not included in our cohort. Previous studies have, however, specifically investigated non-hospitalized patients in the community or in outpatient settings [14,36]. In the community, in patients with HFpEF, age and demographics were similar to our cohort, with patients without PH being slightly younger [36]. In an outpatient setting of patients with chronic heart failure, average age was significantly younger than in our cohort, with no difference in patients without PH [14]. In both settings, PH was associated with worse outcomes. Our cohort corresponds to a real-life representation of non-selected patients hospitalized with ADHF, characterized by comorbidities that often co-exist, and the results may be generalizable and have external validity.

## 5. Conclusions

In patients admitted for ADHF, a high probability of PH as evaluated by echocardiography, was the strongest predictor of all-cause mortality and hospital readmission. LVEF and RVF were not independently associated with prognosis. This suggests that the non-invasive estimation of PH in this setting provides important prognostic information, and its use in routine patient management could lead to improved clinical outcomes.

## Figures and Tables

**Table 1:** Baseline patient characteristics by echocardiographic probability of pulmonary hypertension (PH).

	High Probability of PH (N 117, %))	Low or Intermediate Probability of PH (N 540, %)	*p* Value
Median age, years (range)	78.7 (70.6; 84.6)	78.8 (70.1; 85.0)	0.86
Gender			
Male (%)	69 (59)	310 (57.4)	
Female (%)	48 (41)	230 (42.6)	0.84
BMI	24.7 (21.6; 28.6)	25.6 (22.4; 30.9)	0.02
De novo heart failure (%)	23 (19.7)	179 (33.1)	0.004
Left ventricular function			
Preserved or mid-range (%)	89 (76)	360 (66.7)	
Decreased (%)	28 (24)	180 (33.3)	0.05
Right ventricular function			
Preserved (%)	64 (54.7)	426 (78.9)	<0.001
Reduced (%)	53 (45.3)	114 (21.1)	
Past medical history			
Hypertension	91 (78)	433 (80)	0.59
Diabetes	43 (37)	168 (31)	0.27
CKD	43 (37)	190 (35)	0.8
COPD	22 (19)	76 (15)	0.19
Chronic anemia	56 (48)	213 (39)	0.14
Valvular disease ^a^	46 (39)	162 (30)	0.06
NYHA class			
I	0	7	
II	11	31	
III	33	183	
IV	67	288	0.36
Median systolic pressure at admission (mmHg)	140 (121; 155)	141 (123; 156)	0.43
Median diastolic at admission (mmHg)	80 (70; 88)	80 (70; 93)	0.18
Median heart rate at admission (b/min)	84 (71; 94)	87 (73; 105)	0.04
Median BNP (ng/L) ^b^	805 (444; 2004)	884 (460; 1344)	0.72
Median Pro BNP (ng/L) ^c^	5528 (2729; 10433)	3994 (1702; 8790)	0.08
Median Hb (g/L)	121 (108; 141)	125 (108; 139)	0.63
Median eGFR (mL/min)	50 (35; 53)	51 (36; 70)	0.95

^a^ Defined as moderate or severe mitral or aortic valve stenosis or regurgitation, ^b^ *n* = 113, ^c^ *n* = 544, PH: pulmonary hypertension, CKD: chronic kidney disease, COPD: chronic obstructive pulmonary disease, NYHA: New York Heart Association, BNP: brain natriuretic peptide, and GFR: glomerular filtration rate.

**Table 2:** Medication at admission.

	High Probability of PH (N 117)	Low or Intermediate Probability of PH (N 540)	*p* Value
ACE inhibitors	39 (33.3)	143 (26.5)	0.29
AR antagonists	34 (29.1)	159 (29.4)	1
Loop diuretics	83 (70.1)	265 (49.0)	<0.001
Beta-blockers	61 (52.1)	308 (57.1)	0.47
Calcium channel blockers	29 (24.8)	139 (25.7)	0.92
Antiarrhythmics	9 (7.7)	53 (9.8)	0.67
Antiplatelets	50 (42.7)	237 (43.9)	0.87
Oral anticoagulants	50 (42.7)	199 (36.9)	0.38
Cholesterol lowering	52 (44.4)	245 (45.4)	0.93
Mineral corticoid receptor antagonist	18 (15.4)	66 (12.2)	0.47
Digoxin	9 (7.7)	27 (5.0)	0.39
Oral antidiabetics/Insulin	37 (31.6)	151 (30.0)	0.60
NSAIDs/Corticosteroids	17 (14.5)	42 (7.8)	0.05
Implantable defibrillator	6 (5.1)	39 (7.2)	0.54
CRT device	15 (12.8)	53 (9.89	0.27

PH: pulmonary hypertension, ACE: angiotensin-converting enzyme, AR: aldosterone receptor, NSAIDs: non-steroidal anti-inflammatory drugs, and CRT: cardiac resynchronization therapy.

**Table 3:** Unadjusted analysis of factors associated with all-cause mortality and readmission.

	All-Cause Mortality or Readmission HR (95% CI)	*p* Value
Gender (male)	1.16 (0.96–1.40)	0.12
Age, year	1.00 (0.99–1.01)	0.47
BMI, Kg/m^2^	1.00 (0.98–1.01)	0.65
Comorbidities		
Hypertension	1.29 (1.01–1.65)	0.04
COPD	1.6 (1.25–2.05)	<0.001
Diabetes	1.24 (1.02–1.51)	0.03
Chronic kidney disease	1.49 (1.23–1.80)	<0.001
Chronic anemia	1.45 (1.20–1.75)	<0.001
Echocardiography		
LVEF (<40%) ^a^	0.94 (0.77–1.15)	0.55
Reduced RVF ^b^	1.15 (0.94–1.43)	0.17
High probability of PH ^c^	1.67 (1.33–2.09)	<0.001
Patients with valvular disease ^d^		
Aortic stenosis (*n* = 55)	1.52 (1.10–2.10)	0.01
Aortic regurgitation (*n* = 36)	0.66 (0.42–1.03)	0.07
Mitral stenosis (*n* = 9)	2.67 (1.37–5.20)	0.004
Mitral regurgitation (*n* = 134)	0.94 (0.74–1.19)	0.59
All (*n* = 208) ^e^	1.02 (0.84–1.25)	0.83

^a^ As compared to preserved LVEF, ^b^ as compared to preserved RVF, ^c^ as compared to a low/intermediate probability of PH, ^d^ moderate or severe disease, and ^e^ patients could have more than one valvular disease. LVEF: left ventricular ejection fraction, PH: pulmonary hypertension, BMI: body mass index, and COPD: chronic obstructive pulmonary disease.

**Table 4:** Adjusted association of the probability of pulmonary hypertension, left ventricular ejection fraction, and right ventricular function with primary and secondary outcomes.

	All-Cause Mortality or Readmission HR (95% CI)	*p* Value	All-Cause Mortality HR (95% CI	*p* Value	Cardiovascular Mortality or Readmission HR (95% CI)	*p* Value	Cardiovascular Mortality HR (95% CI)	*p* Value
High probability of PH ^a^	1.67 (1.29–2.17)	<0.001	2.04 (1.38–3.01)	<0.001	1.94 (1.44–2.62)	<0.001	2.7 (1.60–4.57)	<0.001
Reduced LVEF, % ^b^	1.02 (0.81–1.29)	0.84	1.04 (0.72–1.52)	0.81	1.13 (0.86–1.50)	0.37	1.23 (0.73–2.05)	0.43
Reduced RVF ^c^	0.96 (0.76–1.23)	0.77	1.18 (0.81–1.71)	0.40	0.99 (0.74–1.31)	0.93	1.11 (0.66–1.86)	0.70
Aortic stenosis ^d^	1.5 (1.06–2.12)	0.02	1.22 (0.72–2.07)	0.48	1.85 (1.25–2.73)	0.002	2.02 (1.09–3.74)	0.03
Mitral stenosis ^d^	2.37 (1.2–4.66)	0.01	2.95 (1.06–8.26)	0.04	2.20 (0.96–5.03)	0.06	3.38 (1.01–11.30	<0.05
History of COPD	1.47 (1.13–1.91)	0.004	2.62 (1.80–3.81)	<0.001	1.51 (1.10–2.06)	0.01	3.10 (1.89–5.07)	<0.001

^a^ As compared to low/intermediate probability, ^b^ as compared to preserved LVEF, ^c^ as compared to preserved RVF, and ^d^ moderate or severe valvular disease. The complete multivariate model also included chronic kidney disease, chronic anemia, diabetes, hypertension, age and gender. PH: pulmonary hypertension, LVEF: left ventricular ejection fraction, RVF: right ventricular function, and COPD: chronic obstructive pulmonary disease.

## References

[1] Jhund P.S., Macintyre K., Simpson C.R., Lewsey J.D., Stewart S., Redpath A., Chalmers J.W., Capewell S., McMurray J.J. (2009). Long-term trends in first hospitalization for heart failure and subsequent survival between 1986 and 2003: A population study of 5.1 million people. Circulation.

[2] Levy D., Kenchaiah S., Larson M.G., Benjamin E.J., Kupka M.J., Ho K.K., Murabito J.M., Vasan R.S. (2002). Long-term trends in the incidence of and survival with heart failure. N. Engl. J. Med..

[3] Lloyd-Jones D., Adams R., Carnethon M., De Simone G., Ferguson T.B., Flegal K., Ford E., Furie K., Go A., Greenlund K. (2009). Heart disease and stroke statistics--2009 update: A report from the American Heart Association Statistics Committee and Stroke Statistics Subcommittee. Circulation.

[4] Roger V.L., Go A.S., Lloyd-Jones D.M., Benjamin E.J., Berry J.D., Borden W.B., Bravata D.M., Dai S., Ford E.S., Fox C.S. (2012). Executive summary: Heart disease and stroke statistics--2012 update: A report from the American Heart Association. Circulation.

[5] Berra G., Garin N., Stirnemann J., Jannot A.S., Martin P.Y., Perrier A., Carballo S. (2015). Outcome in acute heart failure: Prognostic value of acute kidney injury and worsening renal function. J. Card. Fail..

[6] Ponikowski P., Voors A.A., Anker S.D., Bueno H., Cleland J.G.F., Coats A.J.S., Falk V., Gonzalez-Juanatey J.R., Harjola V.P., Jankowska E.A. (2016). 2016 ESC Guidelines for the diagnosis and treatment of acute and chronic heart failure: The Task Force for the diagnosis and treatment of acute and chronic heart failure of the European Society of Cardiology (ESC)Developed with the special contribution of the Heart Failure Association (HFA) of the ESC. Eur. Heart J..

[7] Rahimi K., Bennett D., Conrad N., Williams T.M., Basu J., Dwight J., Woodward M., Patel A., McMurray J., MacMahon S. (2014). Risk prediction in patients with heart failure: A systematic review and analysis. JACC Heart Fail..

[8] Yancy C.W., Jessup M., Bozkurt B., Butler J., Casey D.E., Drazner M.H., Fonarow G.C., Geraci S.A., Horwich T., Januzzi J.L. (2013). 2013 ACCF/AHA guideline for the management of heart failure: Executive summary: A report of the American College of Cardiology Foundation/American Heart Association Task Force on practice guidelines. Circulation.

[9] Aronson D., Darawsha W., Atamna A., Kaplan M., Makhoul B.F., Mutlak D., Lessick J., Carasso S., Reisner S., Agmon Y. (2013). Pulmonary hypertension, right ventricular function, and clinical outcome in acute decompensated heart failure. J. Card. Fail..

[10] Badagliacca R., Ghio S., Correale M., Poscia R., Camporotondo R., Ferraretti A., Papa S., Pezzuto B., Petrone P., Torre R. (2018). Prognostic significance of the echocardiographic estimate of pulmonary hypertension and of right ventricular dysfunction in acute decompensated heart failure. A pilot study in HFrEF patients. Int. J. Cardiol..

[11] Curtis J.P., Sokol S.I., Wang Y., Rathore S.S., Ko D.T., Jadbabaie F., Portnay E.L., Marshalko S.J., Radford M.J., Krumholz H.M. (2003). The association of left ventricular ejection fraction, mortality, and cause of death in stable outpatients with heart failure. J. Am. Coll. Cardiol..

[12] Owan T.E., Hodge D.O., Herges R.M., Jacobsen S.J., Roger V.L., Redfield M.M. (2006). Trends in prevalence and outcome of heart failure with preserved ejection fraction. N. Engl. J. Med..

[13] de Groote P., Millaire A., Foucher-Hossein C., Nugue O., Marchandise X., Ducloux G., Lablanche J.M. (1998). Right ventricular ejection fraction is an independent predictor of survival in patients with moderate heart failure. J. Am. Coll. Cardiol..

[14] Ghio S., Gavazzi A., Campana C., Inserra C., Klersy C., Sebastiani R., Arbustini E., Recusani F., Tavazzi L. (2001). Independent and additive prognostic value of right ventricular systolic function and pulmonary artery pressure in patients with chronic heart failure. J. Am. Coll. Cardiol..

[15] Kjaergaard J., Akkan D., Iversen K.K., Kober L., Torp-Pedersen C., Hassager C. (2007). Right ventricular dysfunction as an independent predictor of short- and long-term mortality in patients with heart failure. Eur. J. Heart Fail..

[16] Melenovsky V., Hwang S.J., Lin G., Redfield M.M., Borlaug B.A. (2014). Right heart dysfunction in heart failure with preserved ejection fraction. Eur. Heart J..

[17] Meluzin J., Spinarova L., Hude P., Krejci J., Kincl V., Panovsky R., Dusek L. (2005). Prognostic importance of various echocardiographic right ventricular functional parameters in patients with symptomatic heart failure. J. Am. Soc. Echocardiogr..

[18] Meyer P., Filippatos G.S., Ahmed M.I., Iskandrian A.E., Bittner V., Perry G.J., White M., Aban I.B., Mujib M., Dell’Italia L.J. (2010). Effects of right ventricular ejection fraction on outcomes in chronic systolic heart failure. Circulation.

[19] Nochioka K., Querejeta Roca G., Claggett B., Biering-Sorensen T., Matsushita K., Hung C.L., Solomon S.D., Kitzman D., Shah A.M. (2018). Right Ventricular Function, Right Ventricular-Pulmonary Artery Coupling, and Heart Failure Risk in 4 US Communities: The Atherosclerosis Risk in Communities (ARIC) Study. JAMA Cardiol..

[20] Galie N., Humbert M., Vachiery J.L., Gibbs S., Lang I., Torbicki A., Simonneau G., Peacock A., Vonk Noordegraaf A., Beghetti M. (2016). 2015 ESC/ERS Guidelines for the diagnosis and treatment of pulmonary hypertension: The Joint Task Force for the Diagnosis and Treatment of Pulmonary Hypertension of the European Society of Cardiology (ESC) and the European Respiratory Society (ERS): Endorsed by: Association for European Paediatric and Congenital Cardiology (AEPC), International Society for Heart and Lung Transplantation (ISHLT). Eur. Heart J..

[21] Pocock S.J., Ariti C.A., McMurray J.J., Maggioni A., Kober L., Squire I.B., Swedberg K., Dobson J., Poppe K.K., Whalley G.A. (2013). Predicting survival in heart failure: A risk score based on 39 372 patients from 30 studies. Eur. Heart J..

[22] Yancy C.W., Jessup M., Bozkurt B., Butler J., Casey D.E., Colvin M.M., Drazner M.H., Filippatos G.S., Fonarow G.C., Givertz M.M. (2017). 2017 ACC/AHA/HFSA Focused Update of the 2013 ACCF/AHA Guideline for the Management of Heart Failure: A Report of the American College of Cardiology/American Heart Association Task Force on Clinical Practice Guidelines and the Heart Failure Society of America. Circulation.

[23] Aronson D., Eitan A., Dragu R., Burger A.J. (2011). Relationship between reactive pulmonary hypertension and mortality in patients with acute decompensated heart failure. Circ. Heart Fail..

[24] Palazzini M., Dardi F., Manes A., Bacchi Reggiani M.L., Gotti E., Rinaldi A., Albini A., Monti E., Galie N. (2018). Pulmonary hypertension due to left heart disease: Analysis of survival according to the haemodynamic classification of the 2015 ESC/ERS guidelines and insights for future changes. Eur. J. Heart Fail..

[25] Fang J.C., DeMarco T., Givertz M.M., Borlaug B.A., Lewis G.D., Rame J.E., Gomberg-Maitland M., Murali S., Frantz R.P., McGlothlin D. (2012). World Health Organization Pulmonary Hypertension group 2: Pulmonary hypertension due to left heart disease in the adult--a summary statement from the Pulmonary Hypertension Council of the International Society for Heart and Lung Transplantation. J. Heart Lung Transplant..

[26] Rosenkranz S., Lang I.M., Blindt R., Bonderman D., Bruch L., Diller G.P., Felgendreher R., Gerges C., Hohenforst-Schmidt W., Holt S. (2018). Pulmonary hypertension associated with left heart disease: Updated Recommendations of the Cologne Consensus Conference 2018. Int. J. Cardiol..

[27] Vachiery J.L., Adir Y., Barbera J.A., Champion H., Coghlan J.G., Cottin V., De Marco T., Galie N., Ghio S., Gibbs J.S. (2013). Pulmonary hypertension due to left heart diseases. J. Am. Coll. Cardiol..

[28] Vachiery J.L., Tedford R.J., Rosenkranz S., Palazzini M., Lang I., Guazzi M., Coghlan G., Chazova I., De Marco T. (2019). Pulmonary hypertension due to left heart disease. Eur. Respir. J..

[29] Barbieri A., Bursi F., Grigioni F., Tribouilloy C., Avierinos J.F., Michelena H.I., Rusinaru D., Szymansky C., Russo A., Suri R. (2011). Prognostic and therapeutic implications of pulmonary hypertension complicating degenerative mitral regurgitation due to flail leaflet: A multicenter long-term international study. Eur. Heart J..

[30] Simonneau G., Gatzoulis M.A., Adatia I., Celermajer D., Denton C., Ghofrani A., Gomez Sanchez M.A., Krishna Kumar R., Landzberg M., Machado R.F. (2013). Updated clinical classification of pulmonary hypertension. J. Am. Coll. Cardiol..

[31] Hawkins N.M., Virani S., Ceconi C. (2013). Heart failure and chronic obstructive pulmonary disease: The challenges facing physicians and health services. Eur. Heart J..

[32] Iversen K.K., Kjaergaard J., Akkan D., Kober L., Torp-Pedersen C., Hassager C., Vestbo J., Kjoller E., ECHOS-Lung Function Study Group (2008). Chronic obstructive pulmonary disease in patients admitted with heart failure. J. Intern. Med..

[33] Rusinaru D., Saaidi I., Godard S., Mahjoub H., Battle C., Tribouilloy C. (2008). Impact of chronic obstructive pulmonary disease on long-term outcome of patients hospitalized for heart failure. Am. J. Cardiol..

[34] McMurray J.J., O’Connor C. (2014). Lessons from the TOPCAT trial. N. Engl. J. Med..

[35] Omar H.R., Guglin M. (2017). Acute systolic heart failure with normal admission BNP: Clinical features and outcomes. Int. J. Cardiol..

[36] Lam C.S., Roger V.L., Rodeheffer R.J., Borlaug B.A., Enders F.T., Redfield M.M. (2009). Pulmonary hypertension in heart failure with preserved ejection fraction: A community-based study. J. Am. Coll. Cardiol..

